# Bezafibrate mitigates cardiac injury against coronary microembolization by preventing activation of p38 MAPK/NF-κB signaling

**DOI:** 10.18632/aging.205707

**Published:** 2024-10-09

**Authors:** Ruijie Liu, Wenfang Wang, Wenfeng Li

**Affiliations:** 1 Department of Cardiology, Dongguan Songshan Lake Central Hospital, Dongguan 523326, Guangdong Province, China; 2 Department of Cardiology, The First Affiliated Hospital of Ji’nan University, Guangzhou 510627, Guangdong Province, China; 3 Department of Cardiology, Chongyi People’s Hospital, Ganzhou 341399, Jiangxi Province, China

**Keywords:** coronary microembolization, Bezafibrate, myocardial apoptosis, cardiac dysfunction, NF-κB

## Abstract

Coronary microembolization (CME)-induced inflammatory response and cardiomyocyte apoptosis are the main contributors to CME-associated myocardial dysfunction. Bezafibrate, a peroxisome proliferator-activated receptors (PPARs) agonist, has displayed various benefits in different types of diseases. However, it is unknown whether Bezafibrate possesses a protective effect in myocardial dysfunction against CME. In this study, we aimed to investigate the pharmacological function of Bezafibrate in CME-induced insults in myocardial injury and progressive cardiac dysfunction and explore the underlying mechanism. A CME model was established in rats, and cardiac function was detected. The levels of injury biomarkers in serum including CK-MB, AST, and LDH were determined using commercial kits, and pro-inflammatory mediators including TNF-α and IL-6 were detected using ELISA kits. Our results indicate that Bezafibrate improved cardiac function after CME induction. Bezafibrate reduced the release of myocardial injury indicators such as CK-MB, AST, and LDH in CME rats. We also found that Bezafibrate ameliorated oxidative stress by increasing the levels of the antioxidant GPx and the activity of SOD and reducing the levels of TBARS and the activity of NOX. Bezafibrate inhibited the expression of pro-inflammatory cytokines such as TNF-α and IL-6. Importantly, Bezafibrate was found to mitigate CME-induced myocardial apoptosis by increasing the expression of Bcl-2 and reducing the levels of Bax and cleaved caspase-3. Mechanistically, Bezafibrate could prevent the activation of p38 MAPK/NF-κB signaling. These findings suggest that Bezafibrate may be a candidate therapeutic agent for cardioprotection against CME in clinical applications.

## INTRODUCTION

Coronary atherosclerotic unstable plaques may undergo spontaneous erosion, rupture, or fissuring during percutaneous coronary intervention (PCI), and the resulting fragments are washed into distal coronary microcirculation by blood flow, causing CME, which is a common and challenging complication during the perioperative period of PCI clinically [1]. CME may lead to poor local blood supply of the subendocardial myocardium and cause myocardial microinfarction through microvascular vasoconstriction and occlusion, ultimately resulting in the progressive decline of cardiac function and the occurrence of malignant arrhythmias [2]. During reperfusion therapy, patients with CME have a poor prognosis and a high incidence of major adverse cardiac events in the long term [3]. It has been reported that the incidence of CME is about 15-20% in patients with acute coronary syndrome (ACS) during and after PCI, and even up to 45% in high-risk patients [4, 5]. In recent years, with the progression of clinical and basic research, it has been found that myocardial injury caused by CME is closely related to local inflammatory injury, myocardial cell apoptosis, autophagy, and oxidative stress (OS) [6–9]. Skyschally et al. found that the recruitment of inflammatory cells and the increase of inflammatory cytokines such as TNF-α and IL-6 in the myocardial micro-infarction region are the main causes of myocardial contractile dysfunction after CME [10]. Wang et al. found that inhibiting the PTEN gene related to inflammation can alleviate myocardial inflammation after CME in miniature pigs and improve cardiac function [11]. Therefore, multiple genes and signaling pathways related to inflammatory reactions participate in the regulation of myocardial injury after CME. However, the specific mechanism of myocardial injury caused by CME remains to be wholly clarified. Although some measures such as intracoronary application of nitroglycerin, thrombolytics, GPII b/IIIa receptor antagonists, calcium channel blockers, or direct mechanical thrombus aspiration can improve blood flow disorders after CME, the long-term prognosis of patients has not been significantly enhanced [12–14]. Therefore, exploring the pathogenesis of myocardial injury caused by CME through myocardial apoptosis and inflammatory cascade mechanisms may deepen the understanding of CME and provide new ideas and targets for its prevention and treatment.

Peroxisome proliferator-activated receptors (PPARs) belong to the nuclear receptor superfamily of ligand-activated transcription factors [15]. PPAR-α, a subtype of the PPARs, is highly expressed in the heart and can regulate the lipid metabolism homeostasis [16]. Previous studies have found that cardiac deficiency of PPAR-α may result in myosin dysfunction [17]. Bezafibrate (BEZ) is the only drug among marketed drugs that can simultaneously agonize all three subtypes of PPAR receptors. BEZ is mainly used for the treatment of hyperlipidemia [18, 19]. Both clinical and basic experiments have shown that BEZ is beneficial for the prevention and treatment of diabetes and its complications, especially in reducing the risk of cardiovascular diseases [20]. In recent years, BEZ has achieved significant effects in inflammation control [21], antioxidation [22], and anti-apoptosis [23]. Furthermore, BEZ reduces the incidence of myocardial infarction and lowers the risk of cardiac mortality in patients with metabolic syndrome [24]. In a mouse model of Barth syndrome, BEZ showed its beneficial effect on cardiac function [25]. However, the protective effect of BEZ on myocardial injury induced by CME is currently unclear. Here, we aimed to investigate the reparative effect of BEZ on myocardial injury in a CME rat model and explore the underlying mechanism, intending to discover more potential therapeutic strategies for clinical applications.

## MATERIALS AND METHODS

### Animals, modeling, and grouping

48 SD male rats (7-9 weeks) were obtained from Vital River (Beijing, China) and divided into 4 groups (n=12/ each group): Sham group, coronary microembolization (CME), CME+ BEZ (200 mg/kg/day), CME+ BEZ (400 mg/kg/day). The rats were subjected to a 12-h light/dark cycle with unrestricted access to food and water at a constant temperature of 23 ± 2° C. The animal experiment protocols executed in this study were approved by the Institutional Animal Care and Use Committee of Dongguan Songshan Lake Central Hospital. Before the establishment of CME, rats in the CME + BEZ (200 mg/kg/day) and CME +BEZ (400 mg/kg/day) groups were given BEZ at a dosage of 200 mg/kg or 400 mg/kg per day respectively by gavage for 7 days. The dosage of BEZ (Cat#54064ES50, Yeasen Biotechnology (Shanghai) Co., Ltd. China) was chosen based on previous studies [26, 27]. The construction of the CME model was performed following previous studies with minor modifications [28]. After weighing rats, they were anesthetized with 30 mg/kg of 1% pentobarbital sodium injected intraperitoneally. Once rats were in a good anesthetic state, they were fixed in a supine position on a small animal operating table. The hair on the chest was then shaved using an electric trimmer, and the trachea was intubated and connected to a ventilator. After confirming that the respiratory movement of both sides of the chest was normal, the anterior chest wall was disinfected with iodine, and a sterile surgical drape and towel were placed and fixed. The left chest wall from the second to the fifth rib was exposed as the surgical field. The chest wall skin was incised layer by layer along the left edge of the sternum, and the chest muscles were bluntly separated. The third to the fifth rib were cut until the heart was fully exposed. Immediately after opening the chest wall using a retractor, the pericardium was removed by carefully tearing it apart using forceps. The aortic arch at the root of the ascending aorta was clamped with hemostatic forceps, and the heart was lifted using a blunt-tipped small hook. A micro embolus ball was aspirated into an insulin needle, which was then quickly injected into the myocardium through the apex of the heart after which the needle was rotated out. After the aortic arch clamp was released 12 seconds later, the heartbeat was observed. When the heartbeat returned to a normal rhythm, the heart was returned to its original position. After there was no bleeding in the chest cavity or at the injection site of the myocardium, the chest wall muscles and skin were sutured layer by layer. The surgical procedure was performed gently and strictly under aseptic conditions. After closing the incision, the surgical field was disinfected again and wrapped with aseptic gauze. Rats were placed on a postoperative animal warming pad at 37° C to recover and their respiratory changes were closely monitored. When rats resumed spontaneous respiration and could be weaned off the ventilator, the tracheal tube was removed and they were placed in a clean rat cage for rest. In the Sham group, an equal volume of sterile physiological saline instead of the micro embolus ball was injected into the myocardium through the apex of the heart during surgery, and all other surgical procedures were identical.

### The detection of the cardiac function in rats

Cardiac function was detected using an ultrasound instrument from Philip Technologies with parameters including left ventricular ejection fraction (LVEF), left ventricular fractional shortening (LVFS), cardiac output (CO), and left ventricular end-diastolic diameter (LVEDd) [29].

### The detection of the cardiac injury biomarkers in serum

Blood was collected from each animal to achieve the serum, followed by detecting the levels of CK-MB (Cat#ml092665, Shanghai Enzyme-linked Biotechnology Co., Ltd.), AST (Cat#ml092714, Shanghai Enzyme-linked Biotechnology Co., Ltd.), and LDH (Cat#ml095184, Shanghai Enzyme-linked Biotechnology Co., Ltd.) with an automated biochemical analyzer (Beckman Coulter, USA).

### Measurement of OS parameters in cardiac tissues

After sacrificing the animals, the cardiac tissues were collected and the homogenate was obtained, the protein content of which was determined using the BCA method (Cat#P0011, Beyotime, Beijing, China). The GPx (Cat#ml077381, Shanghai Enzyme-linked Biotechnology Co., Ltd. China) level, SOD activity (Cat#ml092619, Shanghai Enzyme-linked Biotechnology Co., Ltd. China), TBARS level (KL-TBARS-Ra, Shanghai kanglang Biotechnology Co., Ltd), and NOX (Cat#ml092596, Shanghai Enzyme-linked Biotechnology Co., Ltd. China) activity in cardiac tissues were determined using the method described by Gholami [30], Beyer [31], Chatterjee [32], and Li [33], respectively.

### ELISA

A 96-well plate was taken out and the standard solution was added to the standard group according to the standard sequence, with distilled water added to the blank control group, and the supernatant collected from centrifugated homogenate of cardiac tissues added to the sample group. Reaction wells were sealed with sealing tape and incubated for 2.5 h. Enzyme conjugate solution was added to the standard and sample groups and incubated with slight shaking for 45 min. After introducing the TMB solution, the sample was cultured for 20 min, followed by adding the stop solution. After achieving the OD value using the microplate reader (MD, USA), the standard curve was drawn, and the concentration of inflammatory cytokines was calculated. TNF-α Elisa kit (Cat#ml002953, Shanghai Enzyme-linked Biotechnology Co., Ltd. China), IL-6 Elisa kit (Cat#ml102828Shanghai Enzyme-linked Biotechnology Co., Ltd. China).

### TUNEL staining assay

Frozen sections of cardiac tissue were prepared with a thickness of 8-12 μm. An appropriate amount of TUNEL reaction solution was added, and the slides were incubated in a 37° C incubator for 30 min. After the sections were air-dried, a freshly prepared DAB staining solution was added. Positive cells were identified by the presence of a brown nucleus. The slides were rinsed with water to terminate the staining reaction. The sections were counterstained with hematoxylin for approximately 3 min. Finally, the slides were dehydrated and sealed with neutral gum. Eight high-power fields (×200) were counted for each specimen, including the positive cells and all other cells. The apoptosis rate (%) was calculated as the number of positive cells divided by the total number of cells ×100%. Each slide was read by two observers and the average value was calculated [34].

### Western blotting assay

The rat cardiac tissue was disrupted with RIPA lysis buffer, and cytosolic and nuclear proteins were extracted. After determining its concentration, the protein was denatured and equivalent samples were loaded onto an SDS-PAGE gel for electrophoresis. The gel was transferred onto a membrane, which was blocked with 5% milk for 2 h. Then, primary antibodies of Bax (1:1500, Cat#ab216494, Abcam, US), Bcl-2 (1:2500, Cat#ab32370, Abcam, US), cleaved caspase-3 (1:1000, Cat##9661, Cell signaling, US), p-p38 (1:800, Cat#4511, Cell signaling, US), p38 (1:2000, Cat#8690, Cell signaling, US), NF-κB p65 (1:3000, Cat#8242, Cell signaling, US), and β-actin (1:2000, Cat#ab8227, Abcam, US) were added and incubated overnight at 4° C. The next day, the membrane was washed with TBST and 1:1000 diluted horseradish peroxidase-labeled secondary antibody (1:3000, Cat#ab288151, Abcam, US) was added and incubated for 2 h. The membrane was washed with TBST and the ECL reagent was added for exposure. The gel was imaged using a chemiluminescent gel imaging system. The grayscale values of the bands were quantified using Image J software [35].

### Statistical analysis

Statistical analysis was performed using SPSS 21.0 software. The results of statistical analysis were expressed as mean ± standard deviation. One-way analysis of variance was used for multiple comparisons between groups. Bonferroni analysis was used as a post-hoc test. P<0.05 was considered statistically significant.

### Data availability

The data is available upon reasonable request from the corresponding author.

## RESULTS

### BEZ improved cardiac function after CME induction

**Figure 1:**
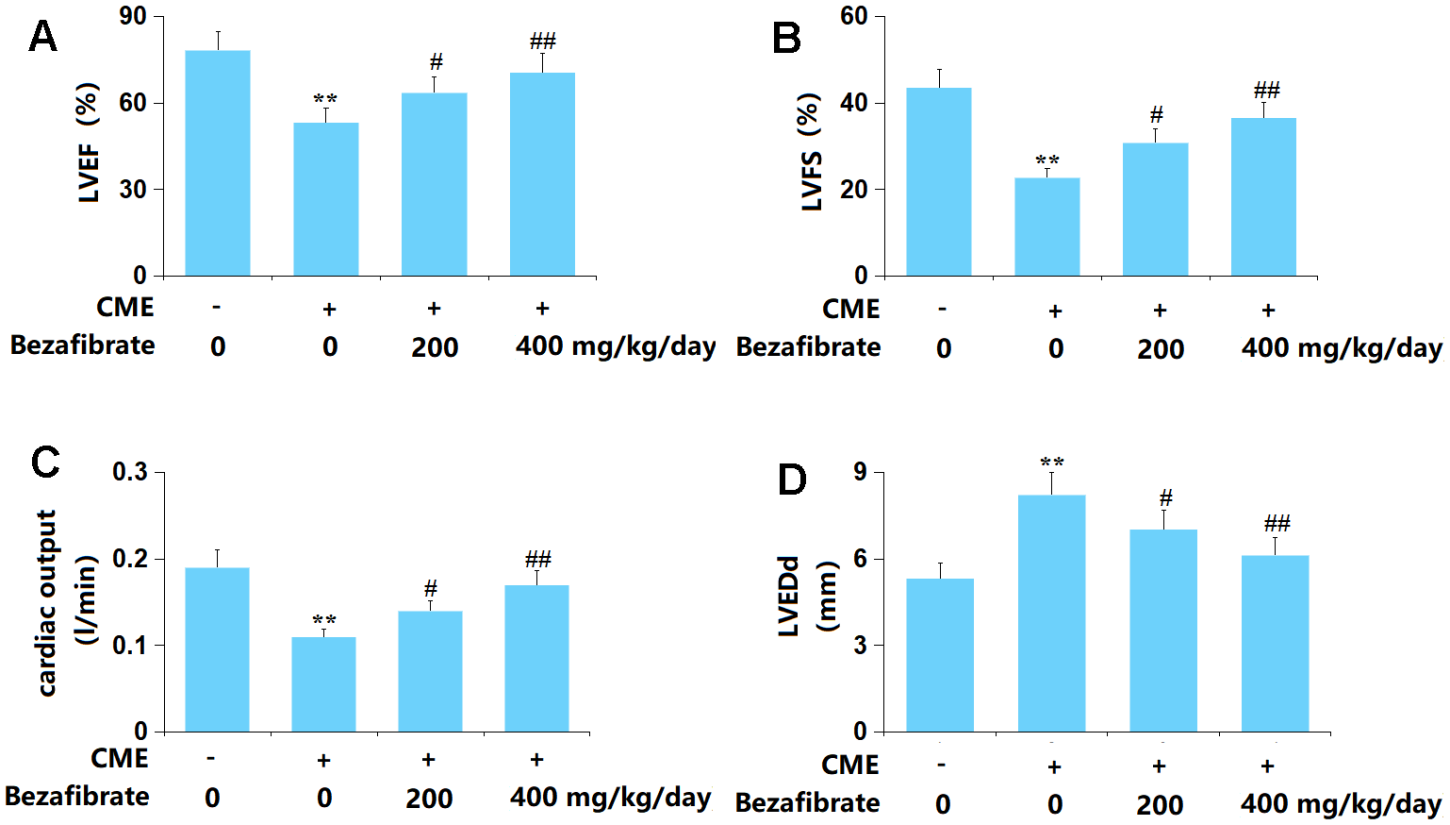
**Bezafibrate improved cardiac function after coronary microembolization (CME) induction.** (**A**) Left ventricular ejection fraction (LVEF); (**B**) Left ventricular fractional shortening (LVFS); (**C**) Cardiac output (CO); (**D**) Left ventricular end-diastolic diameter (LVEDd) (n=12, ^**^, *p*<0.01 vs. control group; ^#^, ^##^, *p*<0.05, 0.01 vs. CME group).

Firstly, the cardiac function of each animal was evaluated. The value of LVEF was markedly reduced from 78.2% to 53.3%, which was reversed to 63.6% and 70.5% by 200 mg/kg and 400 mg/kg BEZ, respectively (Figure 1A). The LVFS values in the Sham, CME, CME+ 200 mg/kg BEZ, and CME+ 400 mg/kg BEZ groups were 43.6%, 22.7%, 30.8%, and 36.6%, respectively (Figure 1B). The CO value was markedly declined in CME rats but sharply elevated by 200 mg/kg and 400 mg/kg BEZ (Figure 1C). Furthermore, the LVEDd value was notably increased from 5.32 mm to 8.21 mm in CME rats then sharply reduced to 7.02 and 6.13 mm by 200 mg/kg and 400 mg/kg BEZ, respectively (Figure 1D). The impaired cardiac function in CME rats was alleviated by BEZ.

### BEZ ameliorated the release of cardiac injury indicators in CME rats

**Figure 2:**
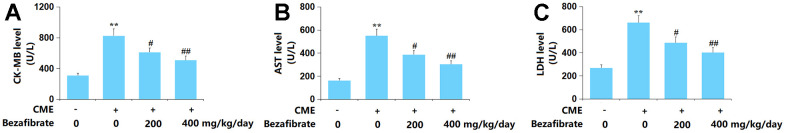
**Bezafibrate ameliorated release of the myocardial injury indicators in CME rats.** (**A**) The CK-MB level; (**B**) The AST level; (**C**) The LDH level (n=12, ^**^, *p*<0.01 vs. control group; ^#^, ^##^, *p*<0.05, 0.01 vs. CME group).

The CK-MB levels in the Sham, CME, CME+ 200 mg/kg BEZ, and CME+ 400 mg/kg BEZ groups were 311.5, 825.8, 611.1, and 508.8 U/L, respectively (Figure 2A). The AST content in CME rats was increased from 163.2 U/L to 551.7 U/L, which was sharply reduced to 387.6 and 305.3 U/L by 200 mg/kg and 400 mg/kg BEZ, respectively (Figure 2B). Moreover, the LDH release in the Sham, CME, CME+ 200 mg/kg BEZ, and CME+ 400 mg/kg BEZ groups was 269.5, 661.6, 487.3, and 403.6 U/L, respectively (Figure 2C). The cardiac injury in CME rats was markedly ameliorated by BEZ.

### BEZ alleviated OS in cardiac tissues of CME rats

**Figure 3:**
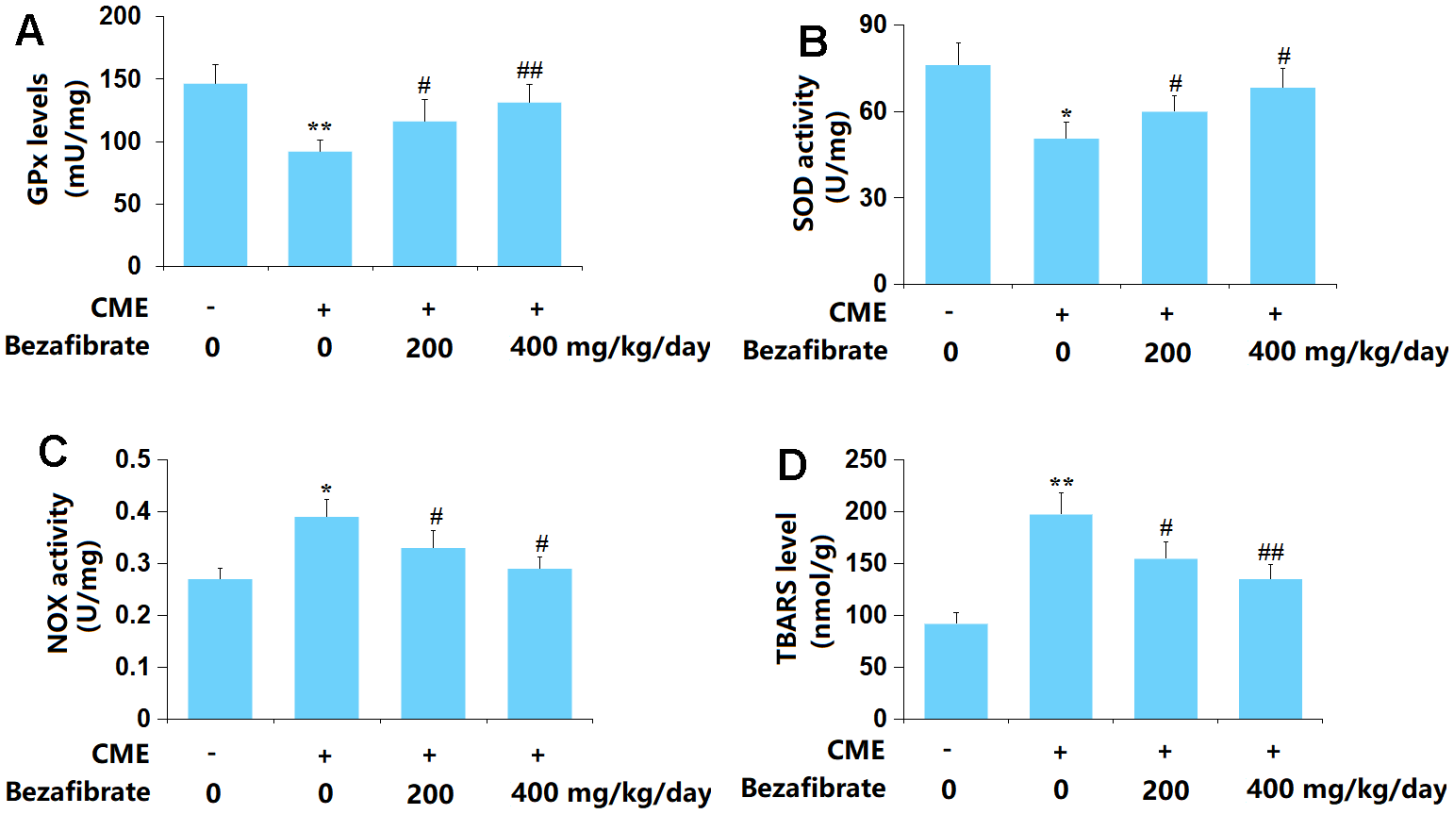
**Bezafibrate ameliorated oxidative stress in cardiac tissues of CME rats.** (**A**) GPx levels; (**B**) SOD activity; (**C**) TBARS level; (**D**) NOX activity (n=12, ^**^, *p*<0.01 vs. control group; ^#^, ^##^, *p*<0.05, 0.01 vs. CME group).

OS is found to participate in the development of CME-induced cardiac injury [36]. The GPx content in cardiac tissues of CME rats was sharply declined but markedly increased by 200 mg/kg and 400 mg/kg BEZ (Figure 3A). Furthermore, the SOD activity was decreased from 76.1 to 50.6 U/mg in CME rats, then markedly elevated to 59.9 and 68.2 U/mg by 200 mg/kg and 400 mg/kg BEZ, respectively (Figure 3B). The TBARS level in the Sham, CME, CME+ 200 mg/kg BEZ, and CME+ 400 mg/kg BEZ groups was 0.27, 0.39, 0.33, and 0.29 nmol/g, respectively (Figure 3C). Moreover, the NOX activity was remarkably increased from 92.1 to 197.6 U/mg protein, which was largely reduced to 154.6 and 135.2 U/mg protein by 200 mg/kg and 400 mg/kg BEZ, respectively (Figure 3D). The OS state evoked in CME rats was repressed by BEZ.

### BEZ inhibited the release of pro-inflammatory mediators

**Figure 4:**
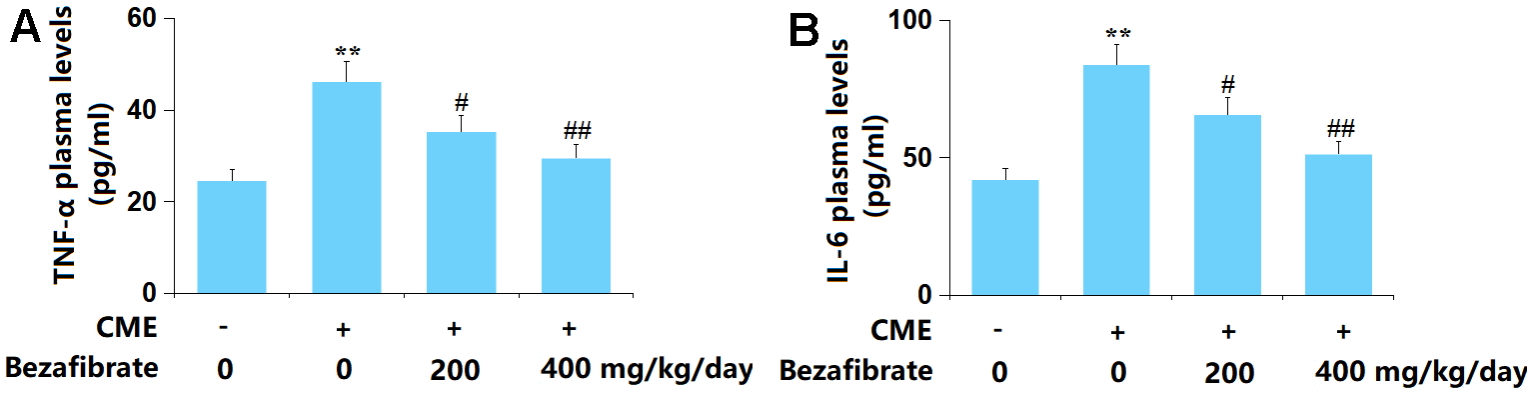
**Bezafibrate inhibited the expression of pro-inflammatory mediators TNF-α and IL-6 against CME.** (**A**) Plasma levels of TNF-α; (**B**) Plasma levels of IL-6 (n=12, ^**^, *p*<0.01 vs. control group; ^#^, ^##^, *p*<0.05, 0.01 vs. CME group).

Enhanced inflammation is one of the main inducers of CME-induced cardiac injury [37]. The plasma TNF-α level in CME rats was largely increased from 24.6 to 46.2 pg/ml, which was remarkably repressed to 35.3 and 29.5 pg/ml by 200 mg/kg and 400 mg/kg BEZ, respectively (Figure 4A). Furthermore, the plasma IL-6 levels in the Sham, CME, CME+ 200 mg/kg BEZ, and CME+ 400 mg/kg BEZ groups were 42.1, 83.8, 65.7, and 51.3 pg/ml, respectively (Figure 4B). The enhanced inflammation in CME rats was alleviated by BEZ.

### BEZ suppressed myocardial apoptosis in cardiac tissues after CME induction

**Figure 5:**
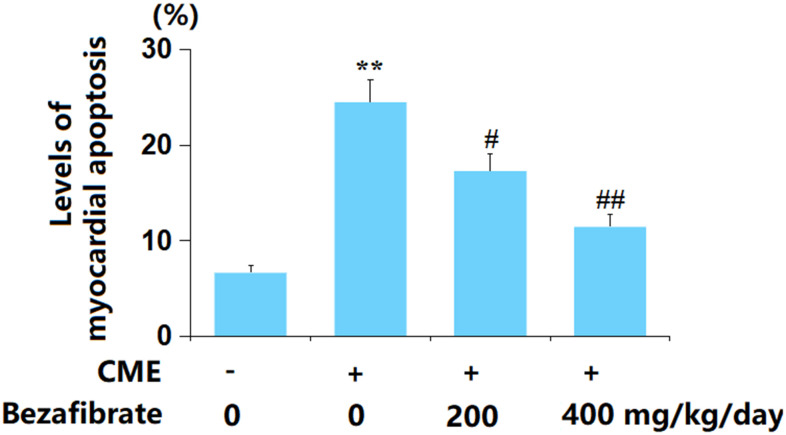
**Bezafibrate suppressed myocardial apoptosis after CME induction.** The levels of myocardial apoptosis were assayed using the TUNEL assay (n=12, ^**^, *p*<0.01 vs. control group; ^#^, ^##^, *p*<0.05, 0.01 vs. CME group).

The apoptosis in myocardial tissues was evaluated using the TUNEL staining assay. The rate of myocardial apoptosis in CME rats was greatly elevated from 6.7% to 24.5%, which was markedly reduced to 17.3% and 11.5% by 200 mg/kg and 400 mg/kg BEZ, respectively (Figure 5), implying an anti-apoptotic property of BEZ in CME rats.

### The effects of BEZ in the expression of Bax, Bcl-2, and cleaved caspase-3

**Figure 6:**
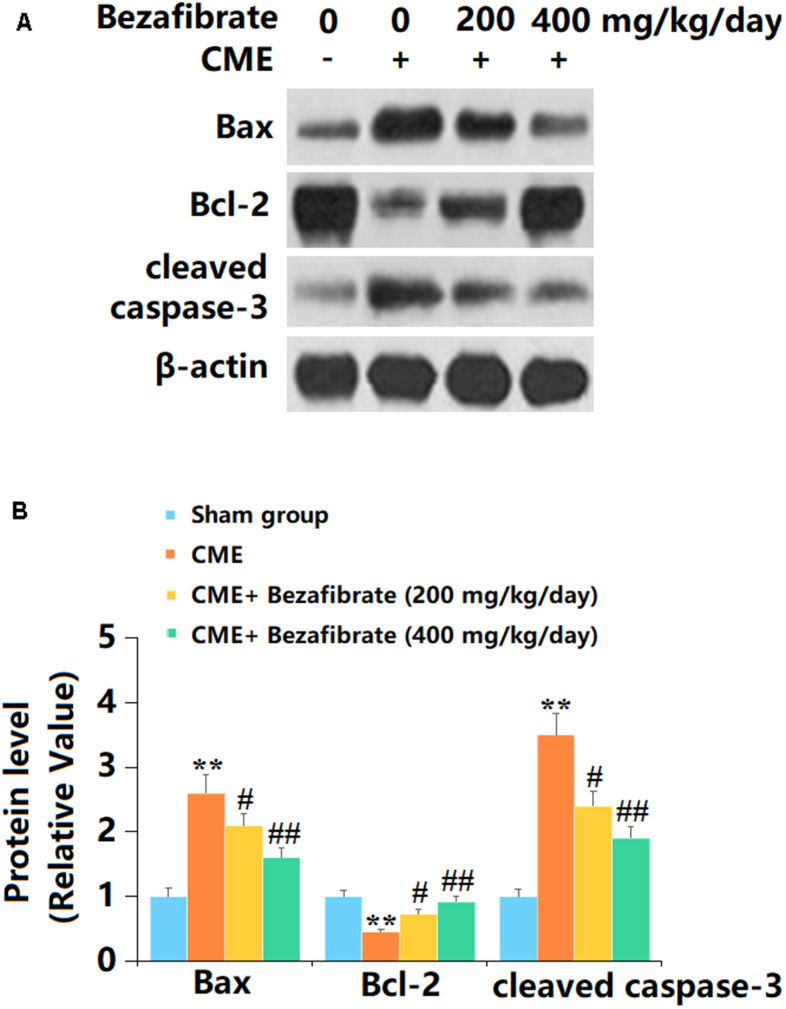
**The effects of Bezafibrate in the expression of Bax, Bcl-2, and cleaved caspase-3 in cardiac tissues of CME rats.** (**A**) Representative images of western blot results of Bax, Bcl-2, and cleaved caspase-3; (**B**) Quantification of Bax, Bcl-2, and cleaved caspase-3 (n=12, ^**^, *p*<0.01 vs. control group; ^#^, ^##^, *p*<0.05, 0.01 vs. CME group).

Subsequently, the levels of apoptotic biomarkers in cardiac tissues were determined. The levels of Bax and cleaved caspase-3 in cardiac tissues were sharply increased, while the Bcl-2 level was markedly decreased in CME rats, all of which were remarkably reversed by 200 mg/kg and 400 mg/kg BEZ (Figure 6A, 6B).

### BEZ prevented activation of p38 MAPK/NF-κB signaling after CME induction

**Figure 7:**
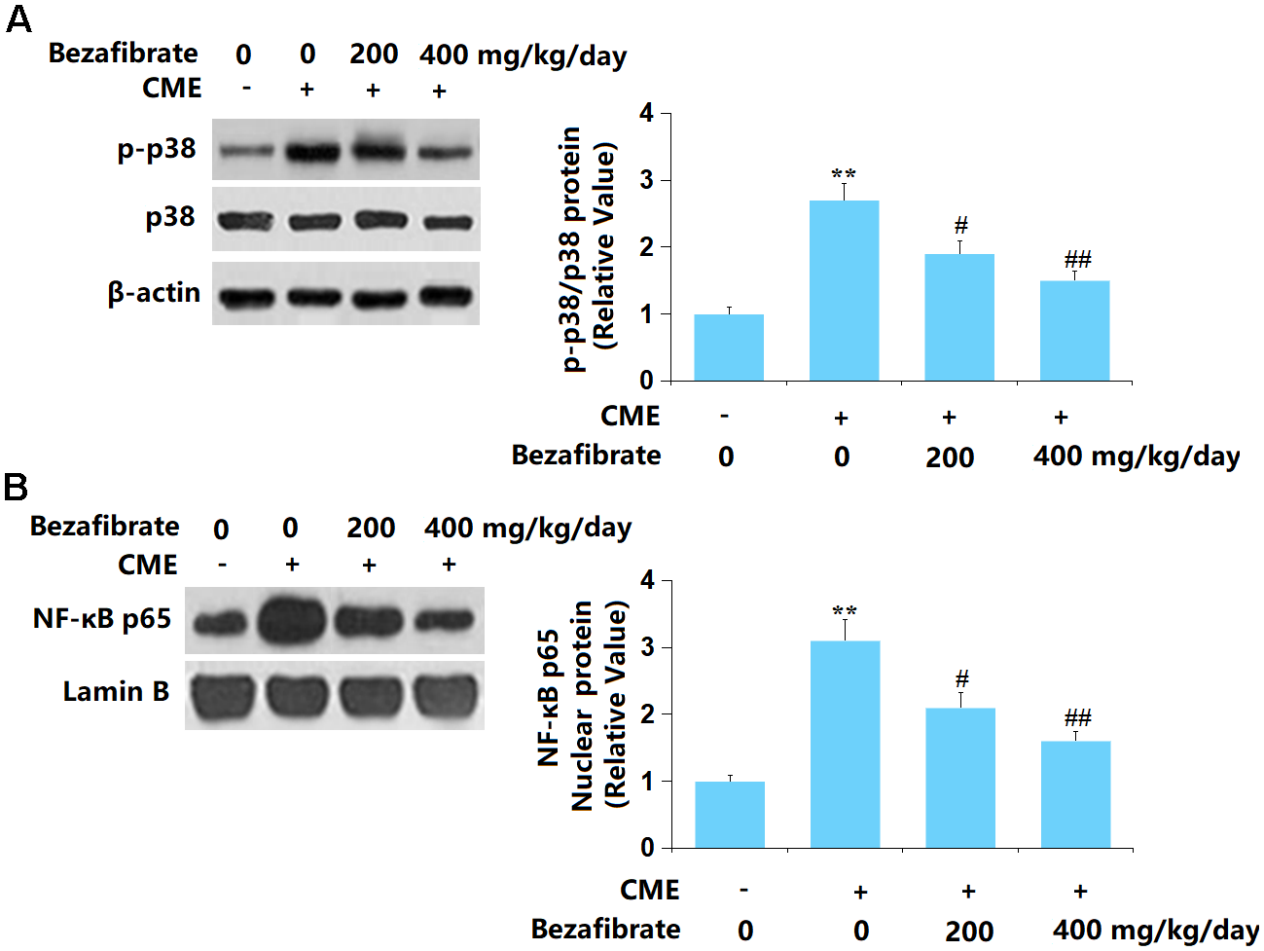
**Bezafibrate prevented activation of the p38 MAPK/NF-κB signaling after CME induction.** (**A**) The levels of p-p38/p38; (**B**) Protein expression of nuclear NF-κB p65 (n=12, ^**^, p<0.01 vs. control group; ^#^, ^##^, p<0.05, 0.01 vs. CME group).

p38 MAPK and NF-κB signaling are claimed to participate in the processing of CME-induced cardiac injury [38, 39]. Herein, the p-p38/p38 and nuclear NF-κB p65 levels were found sharply increased in CME rats but markedly repressed by 200 mg/kg and 400 mg/kg BEZ (Figure 7A, 7B), suggesting a repressive function of BEZ against p38 MAPK/NF-κB signaling in CME rats.

## DISCUSSION

CME reportedly contributes to myocardial injury, which is closely related to the mortality rate of heart failure and the hospitalization rate within one year after PCI treatment in patients with ST-segment elevation myocardial infarction [40]. The inflammatory response following CME has been reported in several studies [41, 42], and it is currently believed that the myocardial inflammatory response caused by CME may be the main cause of myocardial contractile dysfunction. Following the occurrence of CME, a large number of inflammatory cells infiltrate the myocardial microinfarction lesions, accompanied by the release of a large number of inflammatory cytokines, leading to local inflammatory reactions in the myocardium. Moreover, CME causes inflammation not only in and around the microinfarction, but also activates microcirculatory inflammatory reactions, and other parts of the “normal” myocardium show inflammatory factor expression and massive exudation of inflammatory cells [37, 43]. The inflammatory response produces a large number of inflammatory cytokines, such as TNF-α and IL-1β, which cause myocardial injury and induce myocardial contractile dysfunction through various pathways, including promoting myocardial cell apoptosis and expressing adhesion molecules [44]. In addition, several studies have shown that OS significantly participates in myocardial injury caused by CME [36, 45]. Herein, consistent with data presented by Yuan [46], impaired cardiac function and increased release of cardiac injury indicators were observed in CME rats, which were remarkably alleviated by BEZ, suggesting a protective function of BEZ against CME-evoked cardiac injury. Moreover, the aggravated OS state and inflammatory response observed in CME rats were in line with the researches by Xue [36] and Li [47]. Following the administration of BEZ, the OS and inflammation were markedly ameliorated, implying that the function of BEZ might be correlated to the inhibition of OS and inflammation.

CME-evoked myocardial cell apoptosis is one of the reasons for the decrease in heart function [48, 49]. Endogenous and exogenous apoptosis are reported. The endogenous apoptotic pathway, also known as the mitochondrial-mediated cell apoptotic pathway, is initiated by cytochrome C (Cyt-c) released from damaged mitochondria into the cytoplasm [50]. Released Cyt-c binds to apoptosis-related factor 1 (Apaf1), deoxyadenosine triphosphate (dATP), and cysteine aspartate protease 9 (Caspase-9) to form apoptosome, which then activates caspase-3, ultimately leading to the occurrence of apoptotic cascade reactions [51]. Bcl-2 can prevent the release of Cyt-c and reduce Bax binding to the mitochondrial outer membrane, thereby exerting anti-apoptotic effects [52]. SOD activity and MDA content are commonly used to evaluate endogenous antioxidant stress ability and lipid peroxidation degree. When CME occurs, myocardial oxidative free radicals are generated, antioxidant defense enzymes are suppressed, OS is induced, and lipid peroxidation damage occurs [7]. It is claimed that oxidants increase mitochondrial depolarization and induce mitochondrial Cyt-c release into the cytoplasm, further exacerbating apoptotic cell death [53]. Herein, similar to Qin’s report [54], enhanced apoptosis was observed in the cardiac tissues of CME rats, which was notably alleviated by BEZ, further confirming the protection of BEZ against CME-evoked cardiac injury.

P38 MAPK/NF-κB signaling is a critical inflammatory pathway involved in multiple diseases [55–57]. In CME-triggered cardiac injury, the activation of p38 and NF-κB signaling is widely reported [38, 58]. Herein, as presented by other researchers [29, 59], P38 MAPK and NF-κB signaling were markedly activated in CME rats, which were notably repressed by BEZ, implying that the role of BEZ might be correlated to the inhibition of p38 MAPK/NF-κB signaling. In future work, the functional mechanism will be further studied by co-administering BEZ and an agonist of the p38 MAPK/NF-κB axis.

In summary, BEZ alleviated the CME-evoked cardiac injury by repressing OS, inflammation, and apoptosis. These findings suggest that BEZ might be used in the prevention or treatment of cardiovascular complications after CME.
